# Enhanced Terahertz Amplification Based on Photo-Excited Graphene-Dielectric Hybrid Metasurface

**DOI:** 10.3390/nano10122448

**Published:** 2020-12-07

**Authors:** Shengnan Guan, Jierong Cheng, Tiehong Chen, Shengjiang Chang

**Affiliations:** 1 Institute of Modern Optics, Nankai University, Tianjin 300350, China; 2120180997@mail.nankai.edu.cn (S.G.); sjchang@nankai.edu.cn (S.C.); 2 Key Laboratory of Advanced Energy Materials Chemistry (MOE), Institute of New Catalytic Materials Science, School of Materials Science and Engineering, Nankai University, Tianjin 300350, China; chenth@nankai.edu.cn; 3 Tianjin Key Laboratory of Micro-Scale Optical Information Science and Technology, Tianjin 300350, China; 4 Tianjin Key Laboratory of Optoelectronic Sensor and Sensing Network Technology, Tianjin 300350, China

**Keywords:** amplification, metasurface, terahertz

## Abstract

Graphene under optical pump has been shown to be an attractive gain medium with negative dynamic conductivity at terahertz frequencies. However, the amplification over a monolayer graphene is very weak due to its one-atom thickness. In this paper, the proposed graphene-dielectric reflective metasurface effectively improved terahertz field localization and enhanced coherent amplification. The amplification coefficient of 35 was obtained at 3.38 THz at room temperature with an infrared pump intensity of 8 W/mm^2^. As pump intensity increased from 0 to 15 W/mm^2^, we observed a loss–gain–loss transition process, which was discussed in detail through coupled-mode theory. In addition, amplification at different frequencies was achieved by merely re-optimizing the geometric parameters of the dielectric resonators. This study offers an effective solution for enhancing terahertz radiation and developing terahertz lasers.

## 1. Introduction

Terahertz (THz) radiation from 0.1 to 10 THz is the least studied subrange on the electromagnetic spectrum. Many efforts have been devoted to the generation and detection of THz waves, which promotes the rapid development of THz imaging, THz non-invasive detection, and next-generation wireless communication technology [1,2]. Among other types of THz sources, femtosecond laser-driven emitters are the most widely used sources of pulsed THz radiation [3]. When the ultrashort pulse illuminates the photoconductive antenna, the electrically accelerated carriers radiate THz waves [4]. Femtosecond laser-pumped nonlinear crystals, such as ZnTe [5], also give rise to THz radiation based on the optical rectification effect. Currently, the energy conversion efficiency of these emitters is quite low [6], which limits the signal-to-noise ratio of the THz time-domain spectroscopy and hinders long-distance transmission. For these reasons, coherent amplifiers and high-power THz lasers working at room temperature are urgently needed.

Graphene, composed of two-dimensional (2D) carbon atoms arranged in a hexagonal lattice, exhibits many fascinating properties [7,8], including a gapless linear band structure [9,10] and high carrier mobility [11]. Most of its applications fall within the THz frequencies. THz illumination with photon energy, which is less than twice that of Fermi energy (*hν* < 2*E*_F_), is slightly absorbed by graphene due to the intraband carrier transition. In contrast, graphene shows universal absorption of 2.3% from the interband carrier transition when *hν* > 2*E*_F_. Besides the absorption modulation in graphene, the study by Otsuji, T., et al. showed that a sufficiently strong optical pump [12,13] or current injection pump [14] may invert the population in graphene energy bands. As a result of the relaxation time of intraband transitions being much shorter than the recombination time of electron­–hole pairs, the photogenerated electrons and holes are first stimulated to around *E*_F_−*ħω*/2 through carrier–carrier scattering, after which they occupy the energy states closer to the Dirac point through the emission of a series of optical phonons [15,16,17]. This is done within a few picoseconds after the optical pumping and before the electron–hole recombination. Therefore, the conductivity can be driven to a negative value at a specific THz frequency range. Ultrafast carrier dynamics and field amplification have been experimentally observed at THz [11] and infrared frequencies [18], indicating that graphene is a promising gain medium.

However, the negative dynamic conductivity is one or two orders smaller than the characteristic conductivity *e*^2^*/*2*ħ*. The one-atom thickness further limits the interaction of graphene with the THz waves, leading to a negligible amplification effect. The amplification can be enhanced by using multiple layers of graphene [13,19,20], by putting the gain film into the Fabry–Perot cavity or waveguides [9,21], and by combining graphene with plasmonic metasurfaces [22,23,24]. The plasmonic resonators confine the THz waves tightly around the graphene with strong near-field enhancement [25]. However, the Ohmic loss always weakens the amplification. Moreover, only the real part of the graphene conductivity is considered in most of the designs, and the imaginary part is ignored, which may lead to inaccuracies.

In this paper, we studied the enhanced THz amplification when the optically pumped graphene was combined with dielectric metasurfaces. Without material loss, the high-Q resonance of the dielectric resonator together with the back mirror significantly enhanced the field intensity around and outside of the graphene, leading to power amplification coefficient of 35 at room temperature with monolayer graphene. The complex conductivity of graphene was always considered. The transition from absorption to gain, with different pump intensity, was studied in detail based on the coupled-mode theory. The gain frequency was changed by optimizing the geometry of the dielectric resonators.

## 2. Conductivity of Graphene

The dynamic conductivity of graphene under optical pump can be described by [26]:

(1) $$\sigma =\sigma_{\mathrm{int}\mathrm{ra}}+\sigma_{\mathrm{int}\mathrm{er}}$$

(2) $$\sigma_{\mathrm{int}\mathrm{ra}}=\frac{2e^{2}k_{B}T\tau }{\pi \hbar^{2}\left(1+\omega^{2}\tau^{2}\right)}\cdot \ln\left(1+\exp\left(\frac{E_{F}}{k_{B}T}\right)\right)+i\frac{2e^{2}k_{B}T\omega }{\pi \hbar^{2}\left(\omega^{2}+1/\tau^{2}\right)}\cdot \ln\left(1+\exp\left(\frac{E_{F}}{k_{B}T}\right)\right)$$

(3) $$\sigma_{\mathrm{int}\mathrm{er}}=\frac{e^{2}}{4\hbar }\cdot \tanh\left(\frac{\hbar \omega -2E_{F}}{4k_{B}T}\right)+i\frac{e^{2}}{8\hbar \pi }\cdot \ln\left(\frac{{\left(\hbar \omega +2E_{F}\right)}^{2}}{{\left(\hbar \omega \right)}^{2}+{\left(2k_{B}T\right)}^{2}}\right)$$

where *k*_B_ is the Boltzmann constant, *ħ* is the Planck’s constant, *ω* is the electromagnetic wave frequency, *T* is the temperature, and Fermi energy *E*_F_ is related to the pump intensity by

(4) $$E_{F}=6\alpha \frac{v_{f}^{2}}{k_{B}T}\frac{\hbar \tau_{r}\lambda }{\pi c}\cdot I$$

where *α* is the fine-structure constant, *ν*_f_ is the Fermi velocity, and *I* is the pump intensity. Here, the relaxation time τ and the recombination time for electron–hole pairs $\tau_{r}$ were set at 1 ps and 1 ns, respectively. The pump laser wavelength *λ* was 1.5 μm.

**Figure 1:**
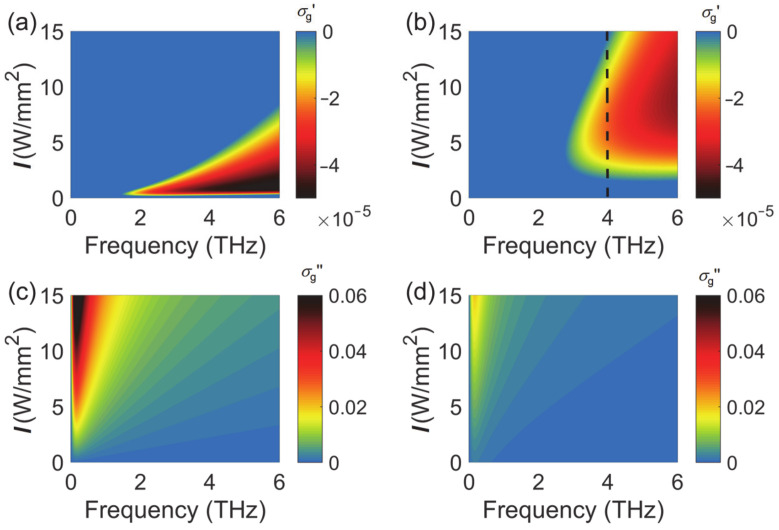
Conductivity of graphene at THz frequencies under optical pump. (**a**) Real part at 77 K. (**b**) Real part at 300 K. (**c**) Imaginary part at 77 K. (**d**) Imaginary part at 300 K. Unit: S.

As one can see from Equation (2), the real part of the conductivity *σ*_intra_ is always positive. The real part of *σ*_inter_ in the Equation (3) assumes negative values when the photon energy is less than twice that of the Fermi energy (*ħω* < 2*E*_F_). The conductivity met this condition when graphene was pumped at high pump intensity I (see Equation (4)). When the negative *σ*_inter_ is large enough to cancel the positive *σ*_intra_, the total conductivity becomes negative. However, with a further increase in pump intensity, *σ*_inter_ tended to saturation and *σ*_intra_ increased. The total conductivity became positive again. Figure 1 displays the real and the imaginary part of the conductivity as a function of pump intensity and frequency at low temperature (77 K) and at room temperature (300 K). For clarity, the positive real conductivity was forced to zero. Graphene exhibited gain characteristics when the real part of the conductivity was negative. At low temperature, the negative conductivity covered a broad frequency range. At room temperature, the negative conductivity moved to higher frequencies with the requirement of larger pump intensity. The magnitude of the real conductivity was in the order of 10^−5^ S. At a fixed THz frequency, such as 4 THz (dash line in Figure 1b), the conductivity experienced a positive–negative–positive transition as the pump intensity increased from 0 to 15 W/mm^2^. There was an optimum pump intensity which corresponded to the largest gain in graphene. In this paper, we mainly focused on the THz amplification at room temperature, where the amplification frequency is between 3 and 6 THz. In addition, if graphene displays a smaller relaxation time, such as 0.1 ps, the negative conductivity moves to a higher frequency (over 6 THz) with the requirement of a slightly higher pump intensity.

## 3. Reflective Amplification in Graphene-Dielectric Structure

### 3.1. Reflective Amplification from Monolayer Graphene

**Figure 2:**
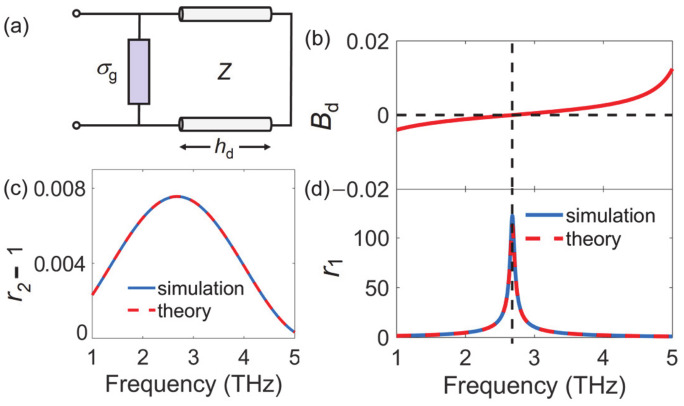
(**a**) The transmission line model of graphene with back mirror. (**b**) Susceptance *B*_d_ of the short-circuited transmission line as a function of frequency. Reflection amplitude as a function of frequency when (**c**) $\;\sigma_{g}=-10^{-5}+0\;i\;S$ and (**d**) $\sigma_{g}=-0.0027+i0\;S$. The solid lines are the COMSOL simulation results, and the dashed lines are the theoretical results.

We first considered a simple structure of optically pumped graphene sheet on top of a back mirror. The graphene and the mirror were separated by a distance of *h*_d_. The transmission line model of this structure is shown in Figure 2a. A thin layer of graphene was equivalent to a shunt admittance $Y=\sigma_{g}=\sigma_{g}^{\prime }+i\sigma_{g}^{\prime \prime }$. The thickness of the spacer layer *h*_d_ (air was considered for simplicity) was set at 28 μm. The transmission line was shorted by the back mirror. The reflection coefficient of the normal excitation can be calculated by [27]:

(5) $$r=\frac{\left(\frac{1}{\eta_{0}}-\sigma_{g}^{\prime }\right)-i\left(B_{d}+\sigma_{g}^{\prime \prime }\right)}{\left(\frac{1}{\eta_{0}}+\sigma_{g}^{\prime }\right)+i\left(B_{d}+\sigma_{g}^{\prime \prime }\right)}$$

Here, *B*_d_ = cos (*kh*_d_)/((tan (*kh*_d_/2) − 2*η*_0_ × sin (*kh*_d_))) is the susceptance of the short-circuited transmission line, whose variation with frequency is shown in Figure 2b. When $\sigma_{g}^{\prime }=-\frac{1}{\eta_{0}}=-0.0027\;$S and $\sigma_{g}^{\prime \prime }=-B_{d}$ are simultaneously met, the reflected beam will be significantly amplified. If we assume the conductivity of graphene to be −0.0027 + 0 i S, the reflection coefficient *r*_1_ from Equation (5) is shown in the Figure 2d by the dashed line. The maximum gain happens when *B_d_* = 0, i.e., the air thickness *h*_d_ = *λ*/4. The peak reflection coefficient is a finite number instead of infinity due to the limited precision of the data points. However, the actual conductivity $\sigma_{g}^{\prime }$ under optical pump is merely around −10^−5^ S (Figure 1), which is two orders smaller than the ideal value. When $\sigma_{g}=-10^{-5}+0\;i$ S is considered, the difference between reflection coefficient *r*_2_ and 1 is the dashed line in Figure 2c. It can be clearly seen that the maximum difference was only 0.008. We further verified the transmission line model using *COMSOL Multiphysics*, version 5.3a; Software for Multiphysics Simulation; COMSOL Ltd., UK, 2017. In this work, we modeled graphene as a two-dimensional Transition Boundary Condition with the surface current density specified by the conductivity in Equation (1). The periodic boundary conditions were applied at the *x* and *y* boundaries and perfect electric conductor (PEC) at the −*z* boundary, while the Floquet port was adopted at the +*z* boundary for both incidence and detection of the electric field *E*_y_. The simulation results perfectly agreed with the theoretical ones in Figure 2c,d. For the above reason, a monolayer graphene cannot offer sufficient gain even with the help of a back mirror.

### 3.2. Enhanced Amplification in Graphene-Dielectric Metasurface

**Figure 3:**
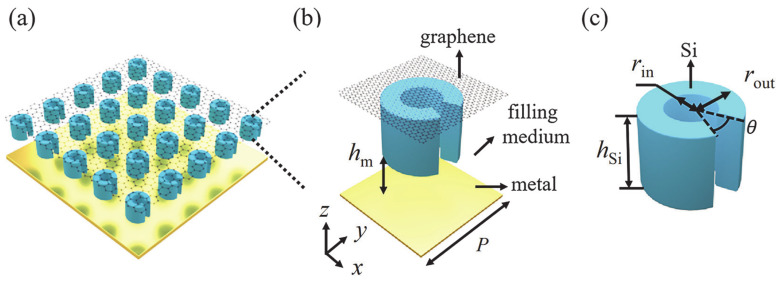
(**a**) Schematic diagram of the proposed graphene-dielectric reflective metasurface. (**b**) The unit cell with material and parameters specified. (**c**) Dimensions of the SRR.

**Figure 4:**
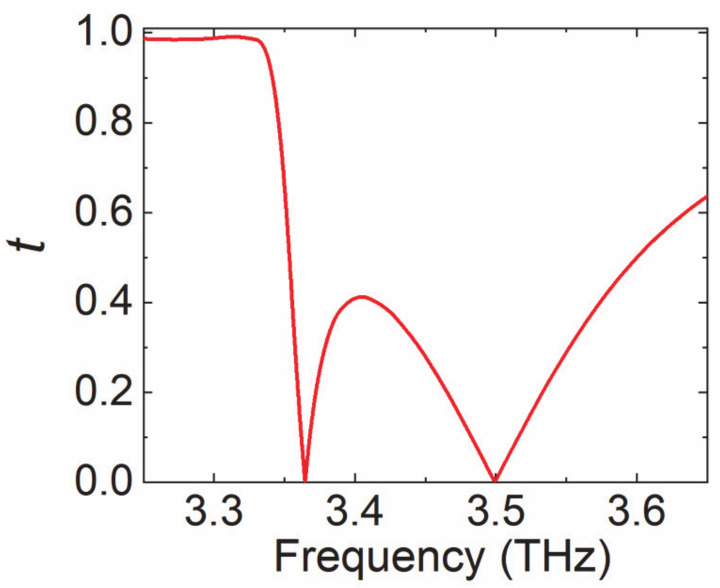
The transmission amplitude of the pure SRR array.

**Figure 5:**
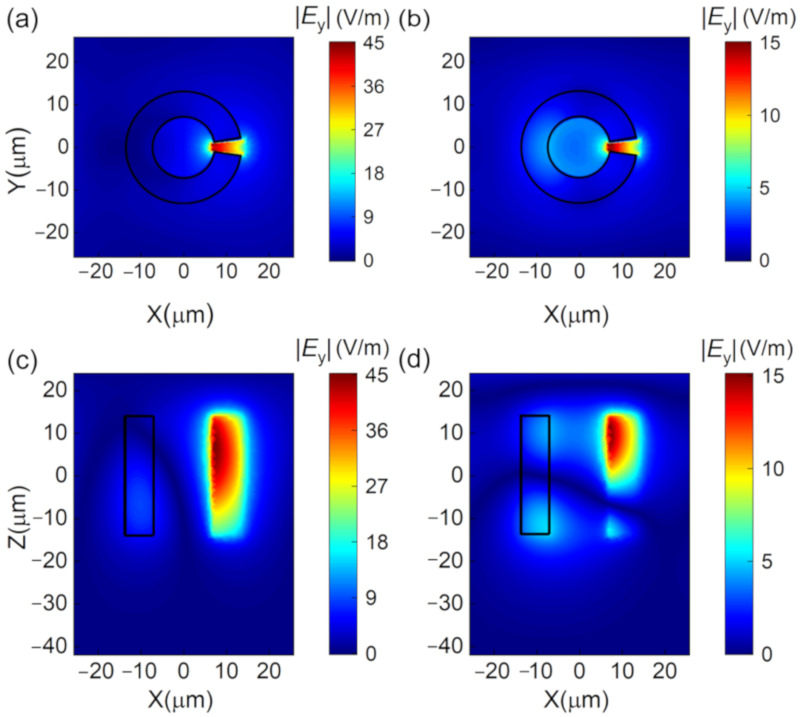
Electric field distribution of SRRs in the *xy* plane at (**a**) *f*_1_ = 3.36 THz and (**b**) *f*_2_ = 3.5 THz. Electric field distribution of SRRs in the *xz* plane at (**c**) *f*_1_ = 3.36 THz and (**d**) *f*_2_ = 3.5 THz.

In order to enhance the interaction between the gain medium and the THz waves, the monolayer graphene was attached to an array of dielectric resonators [28], and they were placed at a distance of 28 μm from the back mirror, as shown in Figure 3a. Figure 3b shows the unit cell with lattice constant *p* = 51.5 μm. The dielectric resonator was a split-ring resonator (SRR) made of silicon, with inner radius *r*_in_ = 6.8 μm, outer radius *r*_out_ = 13.7 μm, thickness *h*_Si_ = 28 μm, and opening angle *θ* = 14°. The opening was symmetric with respect to *y* = 0. The surrounding medium was air and would later be replaced with low-index dielectric toward the end of the study. This graphene-dielectric metasurface was illuminated by *y*-polarization (parallel to the opening angle) plane wave. The transmission responses of the pure SRR array without graphene and the back mirror is investigated in Figure 4. There are two resonances at *f*_1_ = 3.36 THz and *f*_2_ = 3.5 THz. The corresponding near-field distributions at *f*_1_ and *f*_2_ were investigated. The electric fields at both frequencies were well confined in the opening gap of SRR as can be seen in Figure 5a–d. In addition, the field enhancement was much stronger at *f*_1_ than at *f*_2_, as the Q factors of the two resonances were around 170 and 17, respectively. The high-Q resonance at *f*_1_ showed great potential to enhance the interaction of THz waves and graphene once the graphene was attached to the dielectric metasurface.

**Figure 6:**
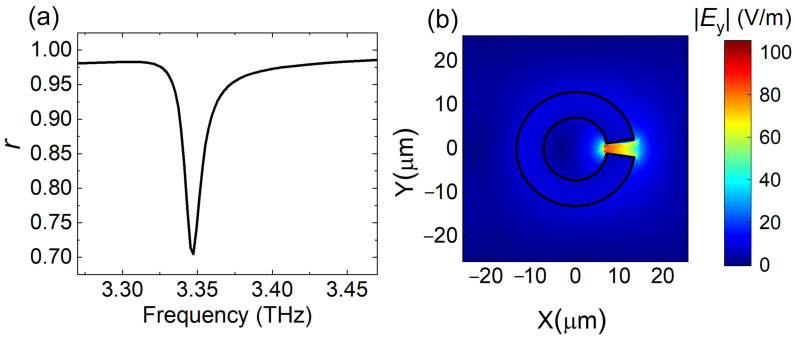
(**a**) Reflection amplitude for graphene-dielectric reflective metasurface as a function of frequency without optical pump. (**b**) Electric field distribution for graphene-dielectric reflective metasurface in the *xy* plane at 3.35 THz (*I* = 0 W/mm^2^).

Next, the graphene was attached to the top interface of the SRR dielectric metasurface as shown in Figure 3, and it was separated with the back mirror by a distance of 28 μm. Without infrared pump, the conductivity of graphene was 17.2 + 167.1 i μS at *f*_1_. Graphene was lossy with positive $\sigma_{g}^{\prime }$. The calculated reflection amplitude of this hybrid metasurface, as shown in Figure 6a, shows that the strong field localization of the SRR enhanced the absorption of graphene at the resonance frequency *f*_1_. The corresponding field intensity at the top interface (Figure 6b) was even stronger than the case of pure SRR. 

**Figure 7:**
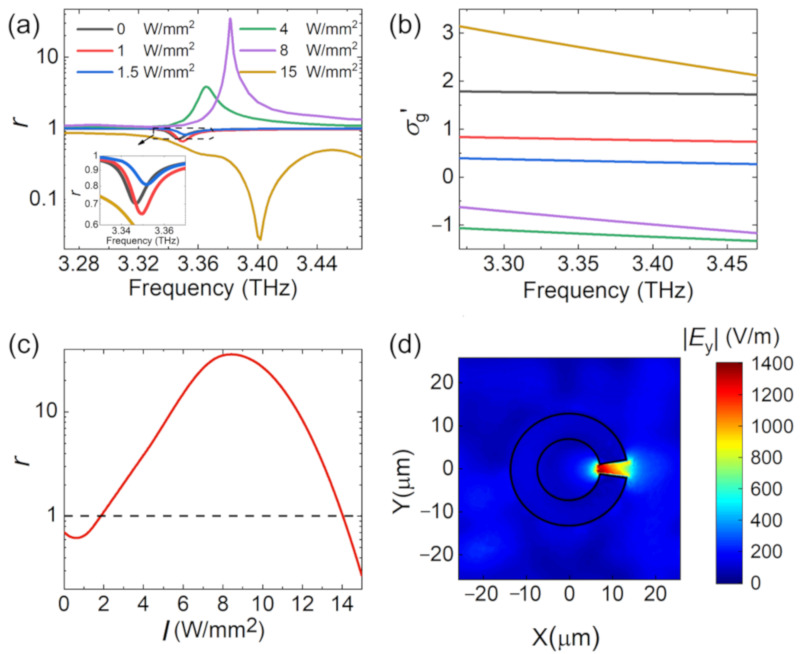
(**a**) Reflection amplitude of graphene-dielectric reflective metasurface under different pump intensities. (**b**) Real conductivity of graphene under different pump intensities. (**c**) The fitting curve for the reflection peak amplitude as a function of optical pump intensity. (**d**) Electric field distribution for graphene-dielectric reflective metasurface in the *xy* plane at 3.38 THz (*I* = 8 W/mm^2^).

The reflection spectrums at different pump intensities are shown in Figure 7a. As the pump intensity increased from 0 to 1 W/mm^2^, we observed increased absorption. As the pump intensity continuously increased, the absorption gradually decreased and became gain at *I* = 4 W/mm^2^ and 8 W/mm^2^, where the conductivity of graphene was negative as shown in Figure 7b. Further increase in the pump intensity pulled the conductivity back to positive values, so that we saw near perfect absorption at *I* = 15 W/mm^2^. Therefore, the reflected THz beam experienced a transition of loss–gain–loss. Variation of the peak reflection amplitude with the pump intensity is plotted in Figure 7c to clarify the loss–gain–loss transition. At the same time, the resonance frequency experienced a blue shift. A maximum amplification coefficient (ratio between the reflection amplitude and the incident amplitude) of 35 was obtained at 3.38 THz when the pump intensity was 8 W/mm^2^. The near-field distribution of the hybrid metasurface at 3.38 THz is shown in Figure 7d. The field intensities in the graphene layer and inside the gap of the SRRs were approximately 14 times larger than that without pump.

**Figure 8:**
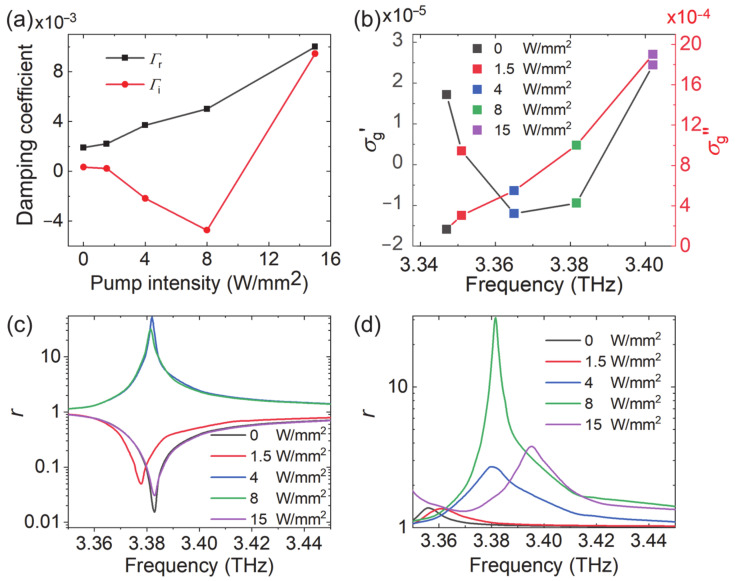
(**a**) The radiation loss $\Gamma_{r}$ and intrinsic losses $\Gamma_{i}$ as a function of pump intensity. (**b**) The conductivity of graphene as a function of pump intensity at different resonance frequencies. Reflection amplitude with different optical pump intensity when the (**c**) imaginary part of conductivity is fixed as 0.001 S and the (**d**) real part of conductivity is fixed as −9.42 μS. The conductivity of graphene is −9.42 + 1000 i μS at 3.38 THz when *I* = 8 W/mm^2^.

To better explain the underlying physics of the non-monotonous variation of the loss and gain, we modeled the metasurface as a one-port resonator. The reflection coefficient r can be derived from the framework of coupled-mode theory (CMT) as [29,30,31]:

(6) $$r=-1+\frac{2\Gamma_{r}}{-i\left(f-f_{0}\right)+\Gamma_{i}+\Gamma_{r}}$$

where *f* and *f*_0_ are the incident wave frequency and resonance frequency, respectively. $\Gamma_{r}$ is the radiation loss due to the radiative decay into free-space electromagnetic waves, and $\Gamma_{i}$ is the intrinsic loss due to material absorption. Perfect absorption happens when $\Gamma_{r}=\Gamma_{i}$, an ideal amplification corresponds to $\Gamma_{r}=-\Gamma_{i}$. Under different pump intensities, the relative values and the sign of the two loss parameters determine the loss/gain characteristic. After numerically fitting the reflection amplitude and phase curves (not shown) using Equation (6), the variation of $\Gamma_{i}$ and $\Gamma_{r}$ with the pump intensity is shown in Figure 8a. The radiation loss $\Gamma_{r}$ increased monotonically as the pump intensity increased, indicating that the resonance had a stronger coupling to free space. On the other hand, the intrinsic losses $\Gamma_{i}$ changed from positive to negative and finally to positive as the pump intensity increased. Figure 8b shows the variation of graphene conductivity with the pump intensity at the corresponding resonance frequency. The variation of $\Gamma_{r}$ was mainly due to the variation of graphene susceptance $\sigma_{g}^{\prime \prime }$. The material loss $\Gamma_{i}$ changed sign in step with the graphene conductance $\sigma_{g}^{\prime }$. At the pump intensity *I* = 8 W/mm^2^, $\Gamma_{i}\;$reached the minimum value −4.725, which corresponded to the maximum amplification at the resonance frequency.

Detailed comparison of Figure 7a,b (or Figure 8a,b) shows that the largest gain happened at *I* = 8 W/mm^2^, although $\sigma_{g}^{\prime }$ was more negative at *I* = 4 W/mm^2^. This is due to the joint effect of the real and imaginary conductivity. Therefore, we separately changed the real and imaginary part of the conductivity to evaluate their contribution. When $\sigma_{g}^{\prime \prime }$ was fixed as shown in Figure 8c, the peak gain happened at *I* = 4 W/mm^2^. When $\sigma_{g}^{\prime }$ was fixed, Figure 8d shows that $\sigma_{g}^{\prime \prime }$ changed the gain magnitude and the resonance frequency. In terms of Equation (6), $\sigma_{g}^{\prime }$ determined $\Gamma_{i}$, and $\sigma_{g}^{\prime \prime }$ changed *f*_0_ and $\Gamma_{r}$. Although $\sigma_{g}^{\prime }$ was more negative at *I* = 4 W/mm^2^, $\sigma_{g}^{\prime \prime }$ at *I* = 8 W/mm^2^ offered a $\Gamma_{r}$ closer to $-\Gamma_{i}$, thus leading to more gain. The blue shift of the resonance frequency in Figure 7a and Figure 8d is consistent, which is due to the variation of the imaginary conductivity.

**Figure 9:**
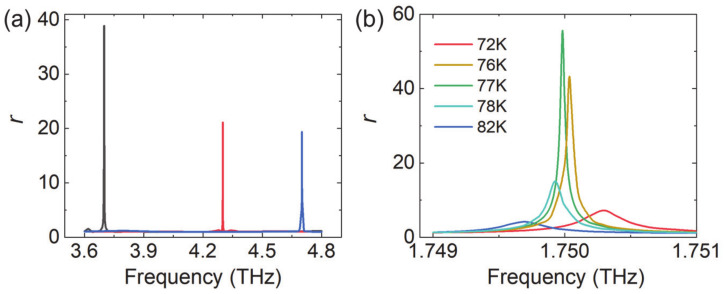
(**a**) Reflection amplitude as a function of frequency when different SRRs are used with pump intensity of *I* = 8 W/mm^2^. (**b**) Reflection amplitude at low temperatures when *I* = 0.35 W/mm^2^.

In the last step, we embedded the silicon SRRs into poly(methyl methacrylate) (refractive index of 1.48) in the reflective metasurface, and kept their top interfaces aligned so that the monolayer graphene could still touch the top interface of SRRs. We optimized the structure to maximize the reflection gain when the pumping intensity was 8 W/mm^2^. The structural parameters were optimized as *p* = 56 μm, *h*_Si_ = 45 μm, and *h*_m_ = 10 μm. Three SRRs with different geometries were selected to enhance the gain at different frequencies. Their dimensions were (1) *r*_in_ = 7.59 μm, *r*_out_ = 12.36 μm (2), *r*_in_ = 10.43 μm, *r*_out_ = 15.96 μm (3), *r*_in_ = 18.29 μm, and *r*_out_ = 23.00 μm. All of them had the same opening angle of 14°. The reflection amplitude as a function of frequency is plotted in Figure 9a. The gain frequency could be regulated from 3.7 THz to 4.7 THz by merely optimizing the radius of SRRs. At the resonance frequency, the reflection amplitude was more than 20 times larger than the excitation. Larger amplification was obtained with larger-Q resonance. In addition, in order to achieve similar gain with lower pump intensity, the metasurface could work at low temperature. As shown in Figure 9b, when *I* = 0.35 W/mm^2^ and *T* = 77 K, an amplification coefficient of 55.6 was obtained at 1.75 THz; after, we re-optimized the lattice constant and radius (*p* = 112 μm, *r*_in_ = 6.56 μm, *r*_out_ = 19.11 μm). As the temperature moved away from 77 K, the gain effect gradually decreased, and the operating frequency also shifted slightly. As mentioned earlier, this is mainly due to the temperature dependence of both the real and imaginary parts of graphene conductivity.

## 4. Conclusions

In summary, we proposed and numerically investigated the amplification of terahertz (THz) radiation based on photo-pumped graphene-dielectric hybrid metasurface. The initial study showed that the monolayer graphene with back mirror was not enough to stimulate sufficient amplification no matter the pump intensity. Graphene was then attached to the high-Q silicon split-ring reflective metasurface, so that the strong field localization enhanced the interaction between terahertz wave and graphene. The amplification coefficient of 35 was obtained at the resonance frequency of 3.38 THz at room temperature with an infrared pump intensity of 8 W/mm^2^. Within the coupled-mode theory, we analyzed the mechanism of loss and gain of the hybrid metasurface with different pump intensities. In addition, THz amplification frequency was widely designable by controlling the split-ring dimensions.
